# Recent advances in surgical techniques for breast reconstruction

**DOI:** 10.1007/s10147-023-02313-1

**Published:** 2023-02-27

**Authors:** Koichi Tomita, Tateki Kubo

**Affiliations:** Department of Plastic and Reconstructive Surgery, Graduate School of Medicine, Osaka University, 2-2 Yamadaoka, Suita, Osaka 5650871 Japan

**Keywords:** Breast reconstruction, Breast implant, Autologous reconstruction, Fat grafting

## Abstract

Although the number of patients with breast cancer continues to rise worldwide, survival rates for these patients have significantly improved. As a result, breast cancer survivors are living longer, and quality of life after treatment is of increasing importance. Breast reconstruction is an important component that affects quality of life after breast cancer surgery. With the development of silicone gel implants in the 1960s, autologous tissue transfer in the 1970s, and tissue expanders in the 1980s, breast reconstruction has advanced over the decades. Furthermore, the advent of perforator flaps and introduction of fat grafting have rendered breast reconstruction a less invasive and more versatile procedure. This review provides an overview of recent advances in breast reconstruction techniques.

## Introduction

In recent years, the number of patients suffering from breast cancer has been on the rise worldwide. In Japan, the cancer statistics in Japan-2022 (Foundation for Promotion of Cancer Research) [1] reported an estimated number of roughly 90,000 people with breast cancer in 2018, with roughly 15,000 deaths. According to the same statistics, breast cancer affects approximately 1 in 11 women at least once in their lifetime. Meanwhile, the survival rate of breast cancer has improved year by year, with a reported 5-year survival of 95.2% for stage I, 90.8% for stage II, 76.3% for stage III, and 35.7% for stage IV [1]. As a result, breast cancer survivors live longer, and their quality of life after treatment has become increasingly important. Breast reconstruction is a key factor in the improvement of quality of life after breast cancer surgery. With the development of silicone gel implants in the 1960s, autologous tissue transfer in the 1970s, and tissue expanders in the 1980s, breast reconstruction techniques have advanced and diversified over the years. This review outlines technical advances in breast reconstruction in recent years.

## Timing of reconstruction

The timing of breast reconstruction is classified into two types: “immediate” and “delayed.” In the former, reconstruction is performed simultaneously with breast cancer surgery, whereas in the latter, reconstruction is performed weeks, months, or years after breast cancer surgery [2]. In immediate reconstruction, advantages include fewer surgeries, better cosmetic results (because the shape of the breast skin envelope is maintained), and avoidance of breast loss experience. However, there are also disadvantages, such as the risk of postoperative skin envelope necrosis, and that postoperative radiation therapy may cause hardening of the reconstructed breast. On the other hand, advantages of delayed reconstruction are that a longer time can be spent in deciding on reconstruction methods, and that there is no need for additional cancer therapy (e.g., radiation after reconstruction), while disadvantages include the need for an increased number of surgeries and hardening of the skin after radiation therapy. These characteristics are summarized in Table 1.

**Table 1:** Characteristics of reconstruction timing

Reconstruction timing	Advantages	Disadvantages
Immediate	Requires fewer surgeries Shape of the breast skin envelope is maintained Patients do not experience loss of breasts	Risk of postoperative skin envelope necrosis Possibility of postoperative radiation therapy Possibility of additional resection due to positive surgical margins
Delayed	More time to consider reconstruction methods No additional cancer therapy (e.g., radiation therapy)	Increased number of surgeries Hardening of skin after radiation therapy

## Abdominal flap

**Fig. 1:**
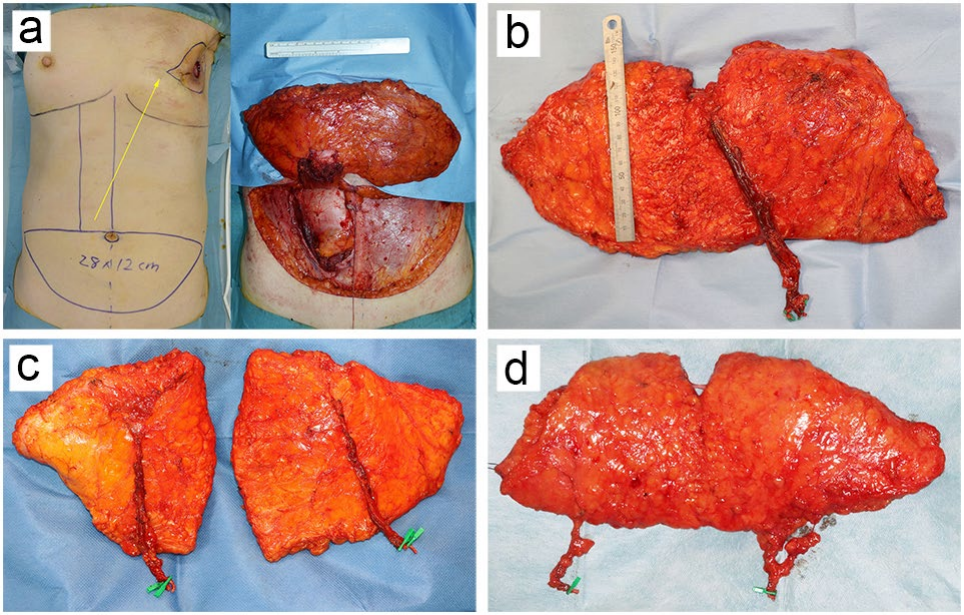
Evolution of the abdominal flap for breast reconstruction. **a** The transversely oriented abdominal musculocutaneous (TRAM) flap is transferred to the chest as a pedicled flap through a subcutaneous tunnel (yellow arrow). **b** The deep inferior epigastric artery perforator (DIEP) flap is raised without sacrificing the rectus abdominis muscle. **c** The DIEP flap is divided into two flaps to reconstruct bilateral breasts. **d** The superficial inferior epigastric artery flap requires no incision of the rectus fascia

The pedicled abdominal flap was first used for breast reconstruction by Robbins in 1979 as a vertically oriented, pedicled musculocutaneous flap [3]. In 1982, Hartrampf et al. described breast reconstruction using a transversely oriented abdominal musculocutaneous (TRAM) flap [4] (Fig. 1a). This method made it possible to create a larger breast shape by effectively utilizing the excess skin and fat of the lower abdomen, while at the same time improving the morphology of the lower abdomen by an abdominoplasty. Later, with the advent of free flap transfer by microvascular anastomoses [5], free TRAM flaps with the deep inferior epigastric artery/vein as a nutrient vessel became common. This method is associated with a flap loss rate as low as 2% and gives much greater freedom of flap placement compared with the pedicled TRAM flap, making it more likely that better esthetic results can be achieved [6].

The free TRAM flap, however, also has a disadvantage: it sacrifices the rectus abdominis, a major muscle, which can lead to complications such as abdominal hernia. In 1989, Koshima and Soeda reported for the first time that a deep inferior epigastric artery skin flap could be raised without sacrificing the rectus abdominis muscle [7]. This skin flap, referred to as the deep inferior epigastric artery perforator (DIEP) flap, led to the development of perforator flaps that also require no sacrifice of the muscle (Fig. 1b). The DIEP flap has gained popularity in the field of breast reconstruction [8–10] and currently represents the most frequently used flap in breast reconstruction. It is especially useful for reconstruction in women with a hereditary risk of breast and ovarian cancer who underwent bilateral risk reducing mastectomy, as it allows for simultaneous bilateral breast reconstruction in which one flap is divided into two (Fig. 1c) [11]. Other breast reconstruction methods using a free superficial inferior epigastric artery (SIEA) flap with the superficial inferior epigastric artery/vein running in the superficial subcutaneous layer as a nutrient vessel have also been reported [12, 13] (Fig. 1d). The SIEA flap requires no incision of the rectus fascia and is even less invasive to the donor site than the DIEP flap. On the other hand, its drawbacks include the short length of the pedicle, and that its use may be limited depending on the degree of vascular development.

## The latissimus dorsi flap

**Fig. 2:**
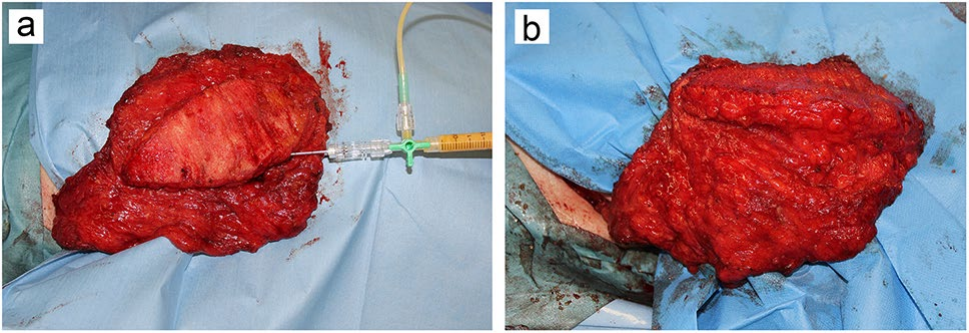
Augmentation of the latissimus dorsi flap with fat grafting. **a** Fat suctioned from the abdomen or thighs is injected into the entire flap tissue. **b** The flap volume is almost doubled

The latissimus dorsi (LD) flap was first described as a myocutaneous flap by the Italian surgeon Tansini in 1906 [14]. The LD flap, however, did not receive much attention in the field of breast reconstruction for many decades afterward, partly because the communication of medical information was limited back then. In the late 1970s, a series of reports on breast reconstruction using the LD flap were published [15, 16]. While the LD flap is a pedicled flap with good circulation and a high degree of freedom, it often lacks the tissue volume necessary for use in breast reconstruction. Due to this disadvantage, the combined use of breast implants was often necessary [17]. In 1987, Hokin and Silfverskiold reported reconstruction without breast implants using an extended LD musculocutaneous flap [18]. Recently, a number of reports were published describing that the volume of the LD flap could be increased by injecting fat suctioned from the abdomen or thighs [19–22] (Fig. 2). With the introduction of this procedure, the LD flap has become a new option for patients who wish to have autologous tissue reconstruction but are not candidates for conventional flap surgery. However, since the sacrifice of the LD muscle (the main muscle involved in shoulder joint motion) is another drawback, there have been attempts to design the thoracodorsal artery perforator flap [23] and muscle-sparing LD flap [24, 25] to preserve the LD muscle.

## Other flaps

In addition to the abdominal flap, perforator flaps from various sites have been developed and used for breast reconstruction. Representative perforator flaps include the profunda artery perforator flap [26, 27], superior/inferior gluteal artery perforator flap [28–30], and lumbar artery perforator flap [31, 32]. These flaps offer alternative options when the abdominal flap or LD flap cannot be used for any reason. The characteristics of each flap are described below.

### Profunda artery perforator flap

Tissue from the posterior thigh vascularized by perforating vessels from the profunda femoris artery and vein is used. The donor-site scar is hidden in the medial posterior thigh. The flap can be harvested bilaterally for bilateral breast reconstruction. However, as the amount of tissue harvested is limited, it is not suitable for the reconstruction of large breasts.

### Superior/inferior gluteal artery perforator flap

Superior and inferior gluteal tissues vascularized by perforating vessels emerging from the superior/inferior gluteal artery and vein are used. The buttocks provide thick adipose tissue and high fibrous connective tissue tension that are suitable for reconstruction of breasts that are not droopy and have a high degree of projection. The amount of tissue that can be harvested from the lower buttocks is greater than that from the upper buttocks. The donor-site scar is concealed by undergarments. The flap can be taken bilaterally for bilateral or large breast reconstruction. On the other hand, its disadvantages include the short pedicle length, the need for changing posture when harvesting, and the asymmetry of the harvested area that may be noticeable when harvested from one side.

### Lumbar artery perforator flap

Perforating vessels from the lumbar artery and vein allow for the use of tissue from the lumbar region, the so-called “love handle” region. Relatively large pieces of tissue can be transplanted and can be harvested bilaterally. On the other hand, similar to the superior and inferior gluteal artery perforator flaps, there are disadvantages such as the short vessel length, the frequent need for vascular grafting, and the need for changing posture when harvesting.

## Prosthetic reconstruction

Reported first by Cronin and Gerow in 1963 [33], silicone gel breast implants have been used in breast reconstruction, primarily delayed reconstruction, after mastectomy. Their use in immediate reconstruction after mastectomy has also been described in the 1970s [34], but it is difficult to obtain good results in cases with large skin excisions. The situation changed in 1982 when Radovan reported the use of a tissue expander [35]. Tissue expanders are placed in the breast after mastectomy and gradually expanded to restore the skin, even in cases with large skin defects. This contributed greatly to expanding the indications of silicone gel breast implants for breast reconstruction.

Since breast implants are artificial, how best to cover the implant is an important issue. In total submuscular coverage, the pectoralis major muscle and serratus muscle are elevated, and the edges of the muscles are sutured together after the implant is placed. Total submuscular coverage is also resistant to the event of skin problems such as mastectomy flap necrosis [36]. There are, however, drawbacks, such as insufficient expansion of the lower pole of the breast and pain during expansion. On the other hand, muscular tightness may be reduced, and the above-mentioned drawbacks may be improved, by using only the serratus fascia to cover the inferolateral region [37]. Recently, the use of scaffolds for total submuscular coverage has been described in many reports. Such scaffolds include absorbable materials and biological materials such as human acellular dermal matrices, and are mainly used for covering the inferolateral portion [38]. This technique increases implant pocket capacity primarily, with an expanded indication for direct-to-implant breast reconstruction after nipple-sparing mastectomy. However, the use of scaffolds reportedly increases the risks of infection and seroma, and thus, caution should be exercised [39]. Moreover, human acellular dermal matrices are currently not available in Japan, as there are no approved products. Recently, prepectoral reconstruction has become more popular; in many cases, the entire implant is covered with a human acellular dermal matrix instead of the pectoralis major muscle, and fat grafting is also used to increase the thickness of the skin envelope [40, 41]. According to previous reports, there is no difference between subpectoral coverage and prepectoral reconstruction in terms of most complications, and a decrease in postoperative pain and animation deformity (from the use of the pectoralis major muscle) is often reported. On the other hand, seroma, rippling, and implant palpability tend to increase. Although data on long-term outcomes are lacking, prepectoral reconstruction may be a useful method. The development of a new scaffold is desirable given the unavailability of human acellular dermal matrices in Japan.

Breast implants currently used in breast reconstruction are mainly classified into the following two types: round implants with a smooth surface, and tear-drop shaped implants with a textured surface [42]. The former provides a softer feeling and lower infection risk than the latter, whereas the latter tends to achieve symmetry more easily in cases of unilateral reconstruction. With regard to patient satisfaction, capsular contracture, implant malpositioning, seroma, or implant failure, previous reports have shown no significant difference between the two types [43, 44]. As for breast implant-associated large cell lymphoma, another important issue [45], almost all reported cases with a medical history of large cell lymphoma involved the use of expanders or breast implants with a textured surface [45]. Although most patients have a favorable prognosis with early detection and timely surgical resection, the chance of a fatal outcome is higher with capsular invasion and tumor bulk. Taken together, breast implants should be chosen while taking into account the advantages and disadvantages of each type in consultation with the patient.

## Fat grafting

**Fig. 3:**
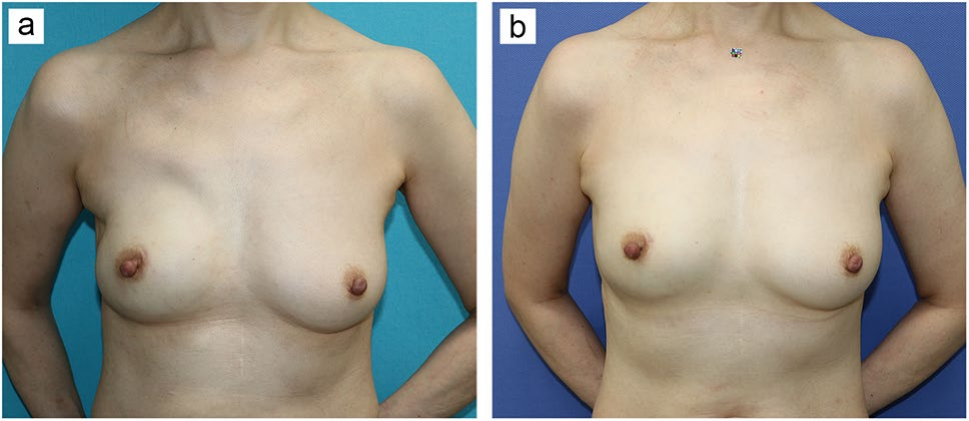
Revision by fat grafting after implant-based breast reconstruction. **a** Tissue insufficiency in the upper chest and axilla is evident seven months after immediate direct-to-implant breast reconstruction following nipple-sparing mastectomy on the right breast of a 51-year-old woman. **b** The reconstructed breast shape is more natural six months after secondary revision surgery with fat grafting into the whole skin envelope

**Fig. 4:**
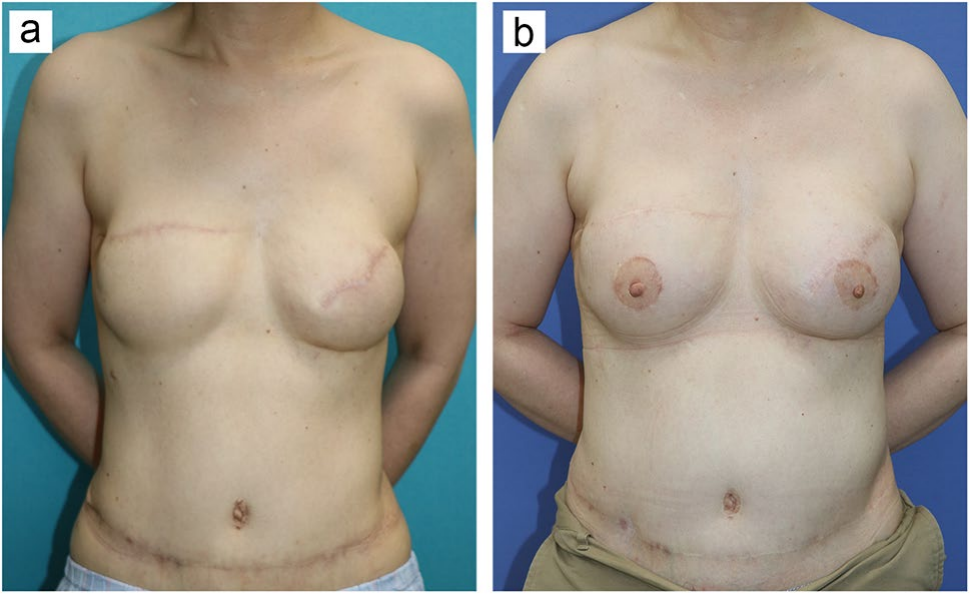
Revision by fat grafting after breast reconstruction with DIEP flaps. **a** Reconstructed breasts are asymmetrical and also lack the natural look 10 months after a 53-year-old woman underwent bilateral breast reconstruction with DIEP flaps. **b** Reconstructed breasts look symmetrical and natural two years after secondary revision surgery with fat grafting into whole breast tissues and reconstruction of the nipple–areola complex

Fat grafting is a technique in which fat tissue is suctioned through an incision a few millimeters in length in the abdomen, thighs, and other areas, purified by removing excess water, blood cells, and oil, and then grafted into the target tissue. Fat grafting is used in breast reconstruction to correct depressed deformities, fill volume deficits, and improve asymmetry. Although the technique itself is not new, over the past decades, technological advances have led to increased safety and efficiency, with expanded indications [46–48]. Retention of a fat graft is greatly influenced by the condition of the graft bed and purified fat, with rates of retention ranging from 20 to 80% [49, 50]. A syringe with a small-diameter cannula is used to inject fat tissue usually less than 2 mm in diameter, known as microribbons, into separate planes [51]. This can result in a good retention rate with reduced local complications such as calcifications and oil cyst formation. In terms of oncologic safety, although most studies are retrospective in nature, there is currently no clinical evidence that fat grafting increases the risk of new breast cancer development, local recurrence, or metastasis [52–54]. In breast reconstruction, specific indications for fat grafting include the use of fat grafts in combination with breast implants. Performing fat grafting in areas that cannot be filled with tissue in cases where breast implants were used alone or for increasing the thickness of the skin envelope reportedly led to improved patient satisfaction and reduced long-term complications [55] (Fig. 3). Fat grafting is also useful for secondary revision after breast reconstruction with autologous tissue such as a free flap (Fig. 4), or secondary revision after breast-conserving surgery. In addition, there are reports of total breast reconstruction using only fat grafting with the use of an external volume expansion device, although multiple sessions of fat grafting are required [51, 56]. However, fat grafting to the breast is not currently covered by insurance in Japan, and various academic societies are taking the lead in efforts to have the procedure covered by insurance.

## Conclusion

Modern breast reconstruction began with breast implants and musculocutaneous flaps, and has since developed further with the introduction of tissue expanders and perforator flaps. More recently, fat grafting, which can be combined with various techniques, has become popular, and options for breast reconstruction have become more diverse. Reconstructive surgeons need to be familiar with these techniques and use them according to the patient’s condition in order to provide breast reconstruction with greater safety and patient satisfaction.

## Data Availability

Data sharing is not applicable to this article as no datasets were generated or analyzed during the current study.
